# An Upper Limb Muscle Fatigue Detection Approach for Overhead Work Using Interface Pressure Complexity Measurement

**DOI:** 10.3390/s26185741

**Published:** 2026-09-09

**Authors:** Guoliang Fan, Shanghua Mi, Majun Song, Jiapeng Yang

**Affiliations:** 1 Hangzhou Innovation Institute, Beihang University, Hangzhou 310052, China; 11725079@zju.edu.cn (S.M.); mjsong1105@126.com (M.S.); 2 School of Mechanical Engineering, Shanghai Jiao Tong University, Shanghai 200240, China; poloerya@sjtu.edu.cn

**Keywords:** muscle fatigue, overhead work, interface pressure, complexity measurement, entropy

## Abstract

**Highlights:**

**What are the main findings?**

- An ergonomic paradigm for overhead musculoskeletal disorders is established to acquire wearable upper limb pressure time series signals.
- A dual-entropy fused complexity measurement method is proposed to extract nonlinear fatigue features for real-time upper limb fatigue detection.

**What are the implications of the main findings?**

- Information entropy detects early muscle fatigue, and sample entropy with better sensitivity and anti-interference capability identifies severe fatigue.
- The dual-entropy fusion method realizes real-time graded upper limb fatigue detection in overhead work for ergonomic safety monitoring.

**Abstract:**

The continuous static contraction of the upper limb during overhead work often leads to progressive muscle fatigue, which is the main cause of work-related musculoskeletal disorders (WMSDs) and reduced operational safety. Traditional fatigue detection methods cannot capture subtle early fatigue characteristics under dynamic working conditions, and sensors are difficult to conveniently deploy in harsh environments. This study constructs an upper limb fatigue detection model based on wearable interface pressure signals and proposes a spatiotemporal dual-entropy fusion strategy. Sample entropy evaluates temporal muscle movement regularity, with a 10% incremental rate-of-change threshold for significant fatigue identification, superior fatigue sensitivity, and anti-interference capability. Information entropy reflects the spatial dispersion of pressure amplitude, adopting a 5% rate-of-change threshold for early fatigue warning. Combined with sEMG comparative verification and nonlinear fitting analysis, a hierarchical monitoring framework is established: single-index abnormality indicates slight fatigue, while dual-entropy synchronous elevation represents severe neuromuscular fatigue. The proposed method overcomes the limitations of single-index evaluation, accurately identifies multiple fatigue levels, and reliably monitors fatigue in real time, providing effective technical support for ergonomic monitoring and occupational safety protection in overhead operations.

## 1. Introduction

Ultra-high voltage (UHV) transmission lines constitute the core framework of the modern power grid, whose stable and safe operation is fundamental to national energy security and reliable power supply [1]. Daily operation and maintenance of UHV transmission lines involve numerous overhead operations, in which operators are required to maintain overhead postures, carry heavy operating tools, and perform repetitive upper limb movements for a long duration [2]. Such high-intensity overhead work conditions and unreasonable biomechanical loading inevitably induce continuous upper limb muscle fatigue [3]. Long-term accumulation of muscle fatigue not only reduces operators’ movement stability and operational accuracy, but also significantly increases the risk of musculoskeletal disorders, constituting a human-factor threat to the safety of UHV line maintenance [4,5,6,7]. In terms of fatigue monitoring and evaluation, traditional methods have prominent technical limitations. The conventional fatigue evaluation system, which mainly depends on subjective questionnaires and macroscopic behavioral observation, cannot realize a quantitative, real-time, accurate perception of muscle fatigue during overhead work [8,9]. Additionally, surface electromyography, another common fatigue detection method, is vulnerable to motion artifacts and requires complex field deployment, further restricting its practical application [10]. Overall, the above defects make it difficult to achieve accurate and continuous monitoring of upper limb fatigue in dynamic scenarios of overhead work [11,12].

As an alternative effective monitoring approach, interface pressure signals can effectively characterize the biomechanical interaction between the human body and operating equipment during overhead tasks, and interface pressure signals contain abundant implicit information on muscle load changes and fatigue evolution [13]. Meanwhile, pressure measurement features simple field deployment and strong anti-interference capability, making it highly suitable for long-term fatigue monitoring of overhead work [14]. In terms of signal analysis, traditional linear indicators fail to capture subtle fatigue changes, while complexity measurement can deeply excavate the dynamic variation characteristics of interface pressure signals, as it is able to sensitively identify subtle dynamic changes in pressure signals induced by early neuromuscular deterioration [15,16]. From the perspective of neurodynamics, fatigue triggers compensatory adjustments in motor-unit recruitment and firing synchronization, neural adaptations that change the spatial distribution and temporal complexity of muscle output, which cannot be fully captured with conventional amplitude-based features. Information entropy and sample entropy quantify such spatial heterogeneity and temporal disorder, providing dynamical markers that reflect the underlying neuromuscular reorganization during muscle fatigue. Their fusion supports multi-dimensional fatigue assessment, mitigating single-index limitations, and further enables threshold-based hierarchical monitoring for early warning and severe fatigue identification for field occupational protection.

## 2. Literature Review

### 2.1. Human Factors and Ergonomics of Overhead Work

Prolonged arm elevation in overhead work triggers upper limb fatigue and safety risks, attracting widespread research attention on fatigue monitoring and modeling [17]. Li et al. [18] validated the prediction performance of shoulder muscle activity models against sEMG data during overhead tasks. The results indicated that model accuracy decreases at high working heights and is further reduced by passive-arm-exoskeleton wearing. Musculoskeletal modeling can simplify exoskeleton testing yet requires optimization to adapt to more complex overhead operation scenarios. Tian et al. [19] explored exoskeleton-induced muscle synergy changes in overhead screwing tasks via sEMG and non-negative matrix factorization. Shen et al. [20] developed a multimodal monitoring wristband that integrates piezoelectric, thermoelectric, and sEMG technologies to collect multiple physiological signals during exercise. The study establishes the correlation between physiological features and exercise intensity, realizing personalized exercise assessment and intelligent health monitoring. Dickerson et al. [21] demonstrated that the adverse impacts of overhead work on musculoskeletal health are modulated by multiple task-related and individual factors, and they verified that optimizing task cycle parameters can effectively improve muscular endurance, with shorter cycle times potentially increasing workers’ endurance duration by up to 25%. Current studies on overhead work-related upper limb fatigue have adopted musculoskeletal modeling, electromyography, and multi-modal wearable devices to analyze muscle activity, muscle synergy alteration, and task factor-driven muscular endurance variation under overhead operation conditions. However, few existing works focus on extracting interface pressure-based features to quantitatively characterize real-time neuromuscular fatigue evolution, especially for practical on-site overhead operation scenarios.

### 2.2. Muscle Fatigue Detection Based on Physiological Signals

With the development of wearable sensing technology, intelligent monitoring of workers’ working status and safety risks has become a research hotspot in industrial ergonomics and occupational safety [22]. In related studies, Semeia et al. [23] compared surface electromyography (sEMG) and mechanomyography (MMG) under two contraction intensities using Lempel–Ziv (LZ) complexity. LZ complexity revealed unique signal characteristics absent from conventional indicators. Earichappan et al. [24] collected synchronous mechanomyography (MMG) and torque signals during repetitive isokinetic knee extensions; the proposed model verifies MMG’s effectiveness for fatigue assessment. Wang et al. [25] collected synchronous sEMG and MMG signals from participants during weight-lifting and extracted the mean power frequency (MPF) features to construct fatigue labeling datasets. Low-cost wearable sensors have good industrial applicability for on-site fatigue monitoring, providing reliable technical support for real-time perception of workers’ physical status during operations [26]. Concha-Pérez et al. [27] realized automatic operational risk assessment and worker behavior identification by adopting inertial measurement and biological signal monitoring technologies. Moyen-Sylvestre et al. [28] investigated muscle fatigue in repetitive low-load tasks via wearable inertial sensors. The power spectrum serves as an effective indicator for muscle fatigue evaluation. Existing research has extensively explored wearable sensor-based muscle fatigue monitoring by adopting sEMG, MMG, and inertial signals, and verified the feasibility of complexity features, power spectra, and statistical indicators for fatigue grading and physical state perception in laboratory scenarios [29,30]. Nevertheless, current monitoring approaches are highly vulnerable to external interferences such as sweat, large-amplitude limb movements, and skin deformation, which reduce the detection accuracy of muscle fatigue and muscle force recognition.

Compared to existing research, the main academic contributions of the present work can be summarized as follows:

- An anti-interference interface pressure signal acquisition and preprocessing method is proposed for overhead assembly scenarios, which improves environmental adaptability compared with traditional sEMG monitoring.
- A dual-entropy fusion fatigue evaluation framework with hierarchical thresholds is established to eliminate single-index errors and achieve accurate progressive fatigue characterization, verified through sEMG-based nonlinear fitting.

## 3. Detection Modeling of Upper Limb Muscle Fatigue

### 3.1. Sensor Layout and Experimental Paradigm

**Figure 1:**
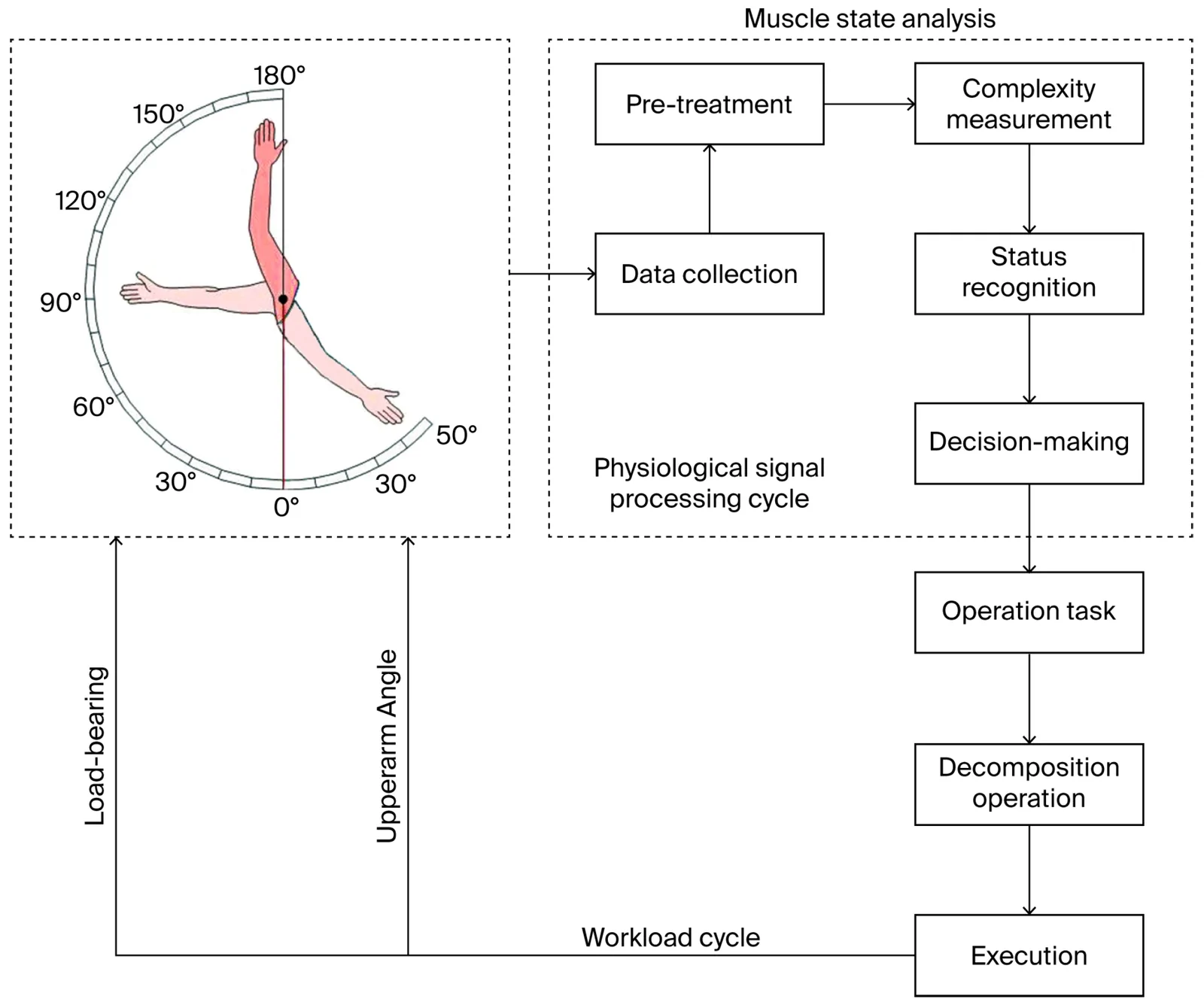
The analytical framework for muscle fatigue states.

Considering that overhead work is the main risk factor leading to shoulder WMSDs, clarifying the fatigue characteristics of upper limb muscles is of great significance for improving work efficiency, thereby reducing workers’ WMSDs [31,32,33]. The identification of work fatigue is often achieved by monitoring key physiological indicators through tools such as electroencephalogram (EEG), which directly reflects the decline in brain alertness by analyzing changes in brain waves. Eye movement indicators, which can effectively capture visual fatigue and drowsiness, are suitable for non-contact monitoring. In addition, surface myography directly detects the changes in electrical signals during muscle activity to assess local muscle fatigue. However, the high-altitude operation scenarios considered here limit the applications of the above technologies. Wearable bands can be easily combined with contact muscle contraction-induced (MCI) force sensors to achieve real-time monitoring and early warning of work fatigue. To monitor the fatigue state of workers’ upper limb muscles during overwork, monitoring armbands are worn on the upper limb. Physiological signal analysis, combined with complexity features, is adopted to identify muscle fatigue states. Figure 1 illustrates the analytical framework for muscle fatigue states.

**Figure 2:**
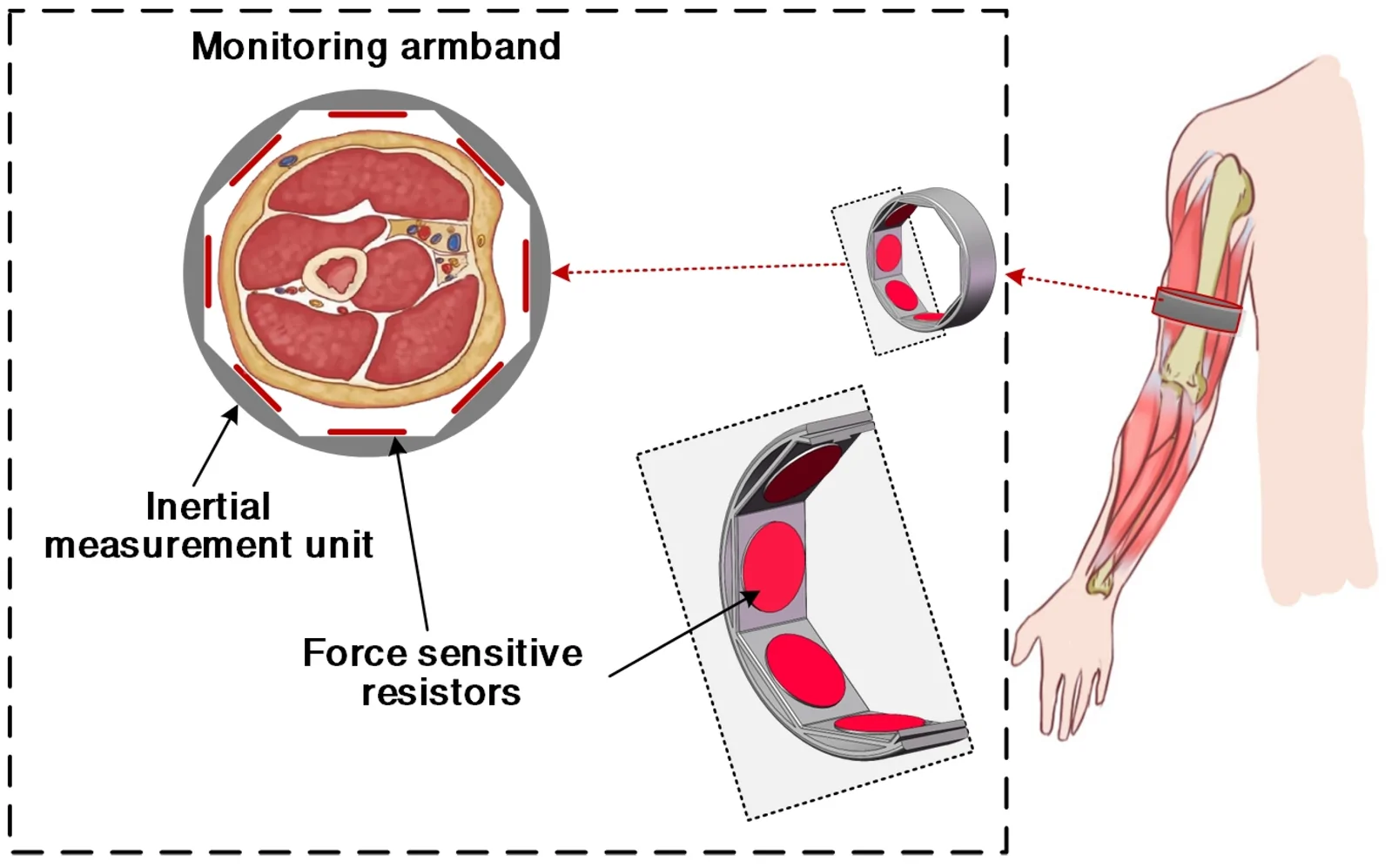
The schematic diagram of sensor layout.

The self-designed wearable signal acquisition device adopts dry, flexible, force-sensitive sensors and integrated microcontrollers as core hardware, independently configured with an 8-channel flexible pressure sensor array at a sampling frequency of 1000 Hz. Sensors are stably attached to target muscle surfaces via elastic straps, and a built-in voltage divider circuit is integrated into the customized hardware structure to convert dynamic surface pressure fluctuations induced by muscle contraction and relaxation into quantifiable voltage signals, achieving non-invasive and real-time monitoring of muscle contraction states and activation intensity. High-quality raw pressure data acquired by the device can effectively support subsequent signal preprocessing and fatigue characteristic analysis for industrial screw assembly scenarios. Eight flexible pressure sensors were arranged annularly, at equal intervals around the upper limb, to measure the interface pressure generated by major upper limb muscle groups during exertion. Due to distinct activation patterns among individual muscles, some sensors yield prominent pressure signals, while others show weak responses. For subsequent analysis, only signals from highly activated sensor channels are focused on characterizing fatigue evolution. Such multi-sensor configuration provides considerable fault tolerance, which guarantees valid signal acquisition, even under complex real-world working postures. Figure 2 illustrates the schematic diagram of the sensor layout.

**Figure 3:**
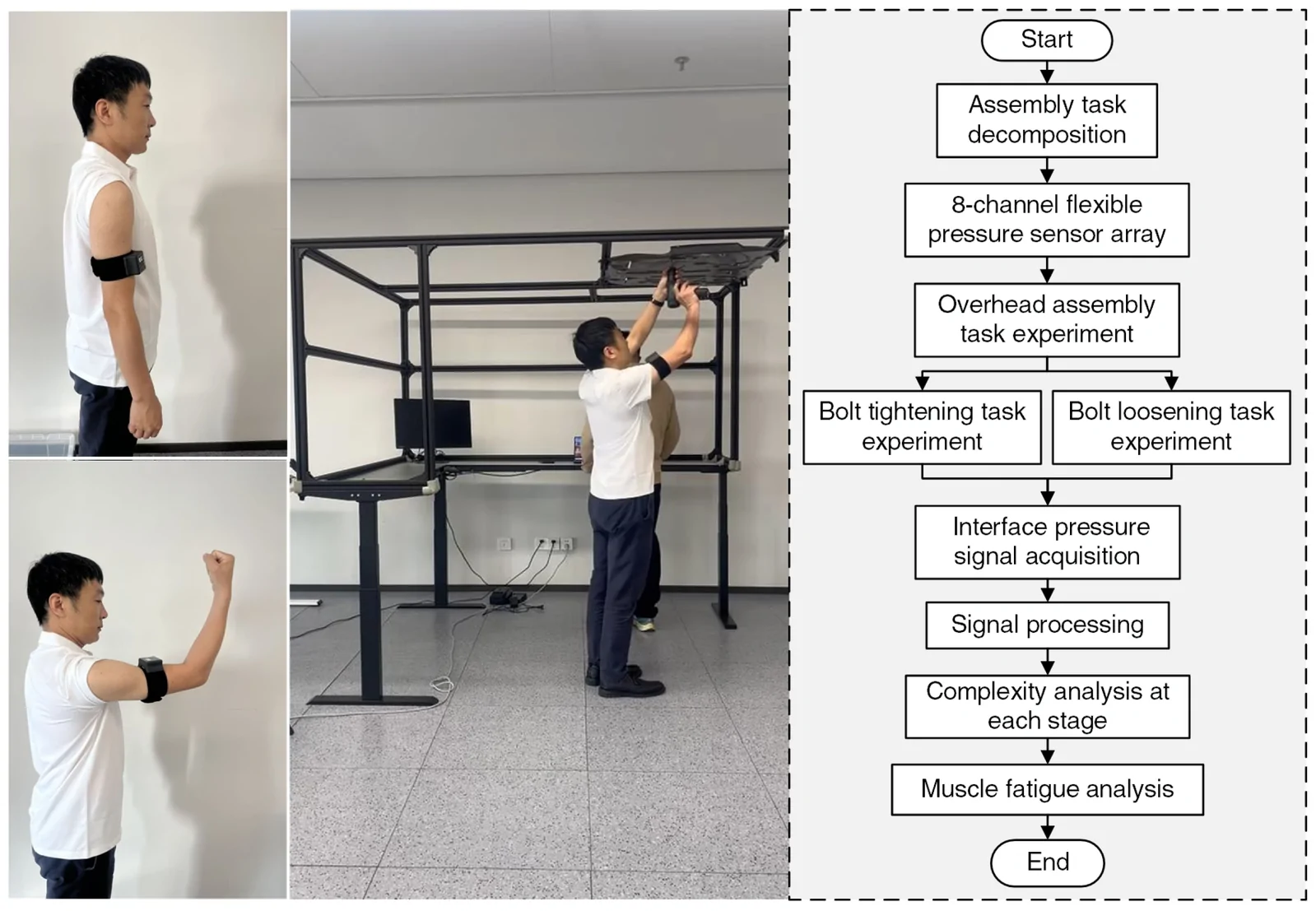
The experimental paradigm and experimental scenario.

The force-sensitive resistor (FSR) sensor is responsible for capturing the local surface pressure changes caused by muscle contraction. The wearable pressure sensor band is wrapped around the upper limb; therefore, the fatigue assessment targets the biceps brachii, triceps brachii, brachialis, and other superficial upper limb muscles, which are persistently activated for elbow stabilization and tool gripping during overhead drill-holding operations. The synchronous collection and fusion processing of dual-channel data are achieved through a microcontroller. The information fusion algorithm is adopted to separate and interpret the signals. The system can identify the movement stages and precisely determine the preparation, transfer, force-application, holding, and ending stages of the movement by using the inertial measurement unit (IMU). By analyzing muscle contraction intensity derived from FSR signals in correspondence with distinct movement phases, the peak contact pressure within each motion stage can be quantified. Discrepancies in pressure distribution profiles between rapid and slow motions can be investigated. Figure 3 illustrates the experimental paradigm and experimental scenario.

The stepwise experimental workflow for analyzing operators’ motion characteristics in industrial screw assembly is elaborated as follows:

***Step 1***: This experiment focuses on typical industrial screw assembly tasks, with the core aim to systematically characterize and quantify operators’ kinematic features under standardized assembly operations.

***Step 2***: Fully complying with on-site standard operating procedures (SOPs), the entire continuous screw assembly movement is split into five sequential, non-overlapping core motion phases: static standby preparation, electric screwdriver retrieval, screw tightening and loosening manipulation, electric screwdriver placement, and returning to the initial standing posture.

***Step 3***: Valid motion datasets associated with screw tightening operations were acquired.

***Step 4***: Subsequent analysis of the collected experimental data was performed to reveal how assembly tasks affect operators’ movement patterns and operational status.

***Step 5***: Comparative analysis of motion data across segmented working phases effectively identifies and summarizes the intrinsic disparities and unique kinematic signatures of movements within each operational stage.

### 3.2. Construction of Dual-Entropy Fusion Complexity Features

A dual-entropy framework is adopted to quantitatively evaluate upper limb muscle working states. The two entropy indices characterize neuromuscular fatigue in mutually independent and complementary temporal and spatial dimensions with different physiological implications. Specifically, sample entropy reflects the temporal complexity of muscle signals to evaluate muscle regulation flexibility and fatigue severity. Higher sample entropy represents more flexible neuromuscular regulation and milder fatigue, whereas lower values indicate solidified muscle control and aggravated fatigue. In contrast, information entropy quantifies the spatial dispersion of interface pressure amplitude, characterizing muscle contraction stability. During overhead operations, cumulative upper limb fatigue deteriorates muscle contraction stability and increases pressure fluctuation, leading to a continuous increase in information entropy. Avoiding the one-sidedness of single-index evaluation, the dual-entropy framework integrates temporal movement regularity and spatial amplitude variability, enabling comprehensive quantitative analysis of muscle control characteristics and fatigue evolution throughout operations; this method provides an effective tool for multi-dimensional, accurate, evaluation of muscle fatigue states.

The core calculation formula of information entropy is as follows:

(1) $$H=-\sum_{i=1}^{B}p_{i}\log_{2}(p_{i})$$

where B is the total number of preset bins, $p_{i}$ represents the occurrence probability of samples in the i−th bin, $p_{i}=n_{i}/N$, $n_{i}$ is the number of samples in the i−th bin, and N is the total number of time series samples. When $p_{i}=0$, the corresponding term is set to 0 without accumulation.

This study adopts the equal-width binning method to discretize continuous interface pressure time series data. Information entropy is used to quantify pressure amplitude dispersion and muscle contraction stability. The algorithm takes one-dimensional time series data and preset bin number as inputs, outputting the corresponding information entropy value. The algorithm is provided in Appendix A (Algorithm A1). The specific steps are as follows:

***Step 1***: Parameter initialization. Read the time series data, calculate the maximum and minimum values of the data, and compute the equal-width bin interval based on the preset bin number.

***Step 2***: Data binning and frequency statistics. Initialize the frequency of each bin to zero. Traverse all-time series data, calculate the bin index of each data point, correct boundary overflow, then count the number of samples in each bin.

***Step 3***: Probability distribution calculation. Calculate the sample probability of each bin, according to the bin frequency and total sample size, and establish the data probability distribution model.

***Step 4***: Information entropy calculation. Traverse all bin probabilities and exclude zero-probability items. Accumulate results based on the base-2 logarithmic entropy formula to obtain the final information entropy of the time series data.

***Step 5***: Result output. Return the calculated information entropy value to characterize the amplitude dispersion of interface pressure signals and the stability of muscle contraction.

The core calculation formula of sample entropy is as follows:

(2) $$\mathrm{SampEn}(X,m,r)=-\ln(\frac{A}{B})$$

where m denotes the embedding dimension, r denotes the similarity tolerance threshold, B is the total number of effective template matches with embedding dimension m, and A is the total number of effective template matches with embedding dimension m+1. The formula is valid only when both A and B are greater than 0; otherwise, the sample entropy is defined as NaN.

The sample entropy algorithm is used to calculate the complexity of preprocessed interface pressure time series signals, quantifying the dynamic regulation ability and fatigue level of upper limb muscles. Taking time series data, embedding dimension and tolerance threshold as inputs, the algorithm outputs the final sample entropy value. The algorithm is provided in Appendix A (Algorithm A2). The specific steps are as follows:

***Step 1***: Define the template matching counting function. Input the time series, embedding dimension m and tolerance threshold r. Traverse all valid template vectors, calculate the Chebyshev distance between vector pairs, and count the total number of matching samples whose distance is less than the threshold.

***Step 2***: Count matching quantities hierarchically. Call the template matching function with embedding dimension m and m+1, respectively, and obtain the matching count B under dimension m and matching count A under dimension m+1.

***Step 3***: Calculate sample entropy. If both A and B are greater than 0, the sample entropy is calculated with the formula SampEn=−ln(A/B). Otherwise, the result is set to NaN as invalid data.

***Step 4***: Output result. Return the final sample entropy value to characterize the temporal complexity of interface pressure signals, muscle regulation flexibility, operational fatigue level.

Algorithmically, information entropy partitions signal amplitude into discrete bins to calculate value-occurrence probability, which only reflects amplitude distribution and discards temporal order information. Sample entropy, instead, reconstructs time series templates via embedding dimension and counts template matches using Chebyshev distance, thereby evaluating temporal regularity of signal evolution. Therefore, information entropy captures spatial dispersion of pressure magnitude, while sample entropy tracks temporal disorder of muscle motion. This joint utilization addresses the intrinsic deficiency of single-index measurement and enables multi-dimensional fatigue quantification.

## 4. Experimental Validations

### 4.1. Data Preprocessing

Screw assembly is a typical basic operation in the industrial field. The upper limb muscle control states and fatigue levels of operators directly determine the accuracy, efficiency, and safety of assembly operations. Interface pressure signals can intuitively reflect the contraction and relaxation characteristics of upper limb muscle groups, representing effective indicators for characterizing muscle control performance and evaluating operational fatigue. To unify the experimental standards, the complete screw assembly operation is standardized into five consecutive core stages, including standing still at rest, picking up the electric drill, tightening screws, setting down the electric drill, and returning to the standing posture. Standardized wearing procedures were implemented to ensure experimental repeatability. For each participant and every repeated trial, the armband was tightened to a fixed scale mark on the strap, achieving close upper limb fitting, without excessive local compression, to establish a unified experimental framework for subsequent segmented signal processing and refined analysis.

**Figure 4:**
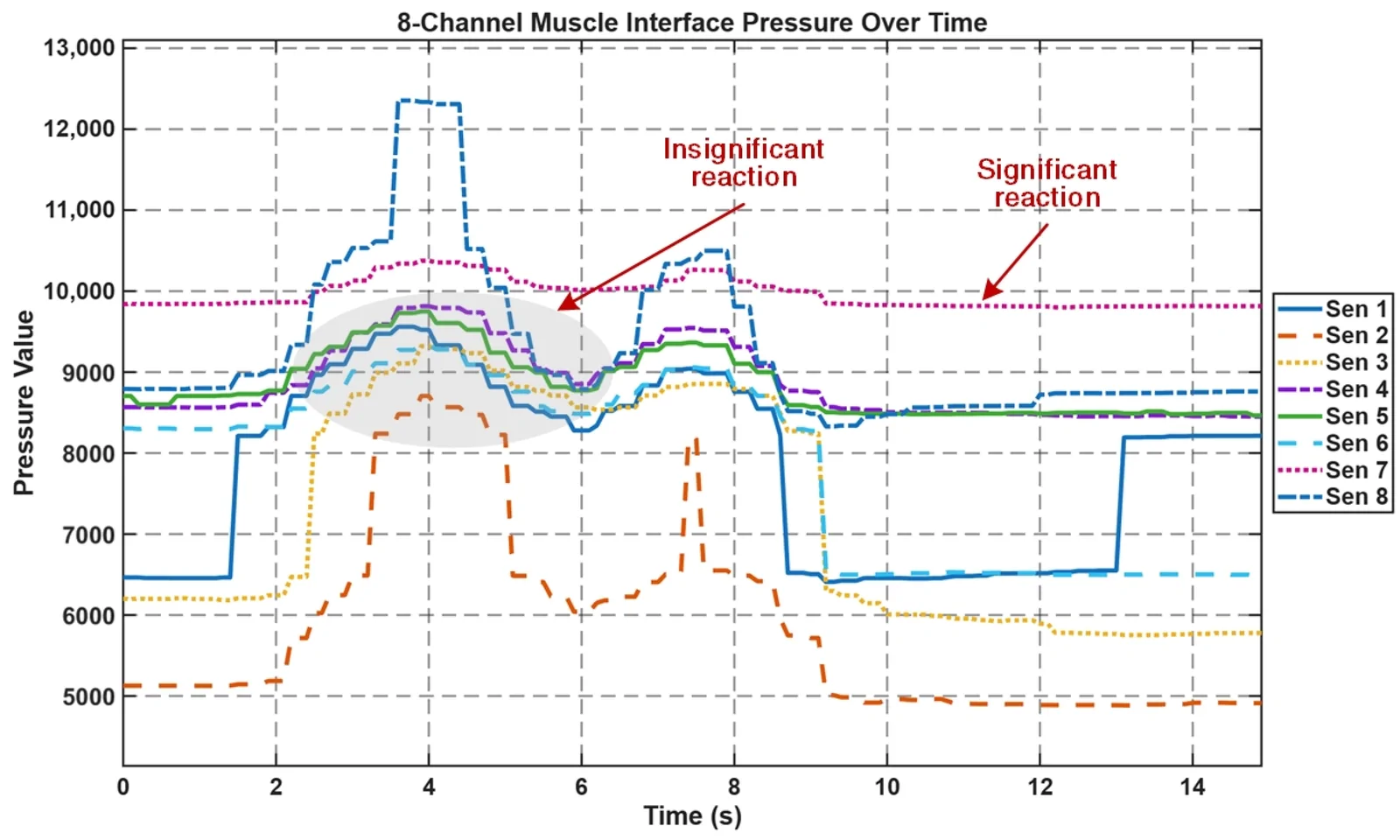
The pressure signals of the upper limb muscle groups.

A total of 10 healthy adult males with no upper limb muscle injuries or fatigue-related chronic diseases and normal visual and limb motor functions were recruited as participants; all were required to refrain from strenuous upper limb exercise within 24 h before the experiment to ensure a consistent initial muscle state. Each experimental condition was repeated for six valid trials per participant. The average of valid-trial data was used for subsequent analysis to mitigate experimental interference arising from cumulative residual fatigue. Figure 4 illustrates the pressure signals of the upper limb muscle groups.

Relying on the high fault tolerance of this annular layout, precise one-to-one alignment between each sensor and target muscle was not mandatory; such an arrangement reduced the susceptibility of signal acquisition to motion artifacts and enabled convenient practical deployment during overhead operations. Affected by heterogeneous muscle-activation patterns across regions, some sensor channels produce prominent responses, while others exhibit weak signals, which accounts for the distinct channel-to-channel differences observed in Figure 4.

**Figure 5:**
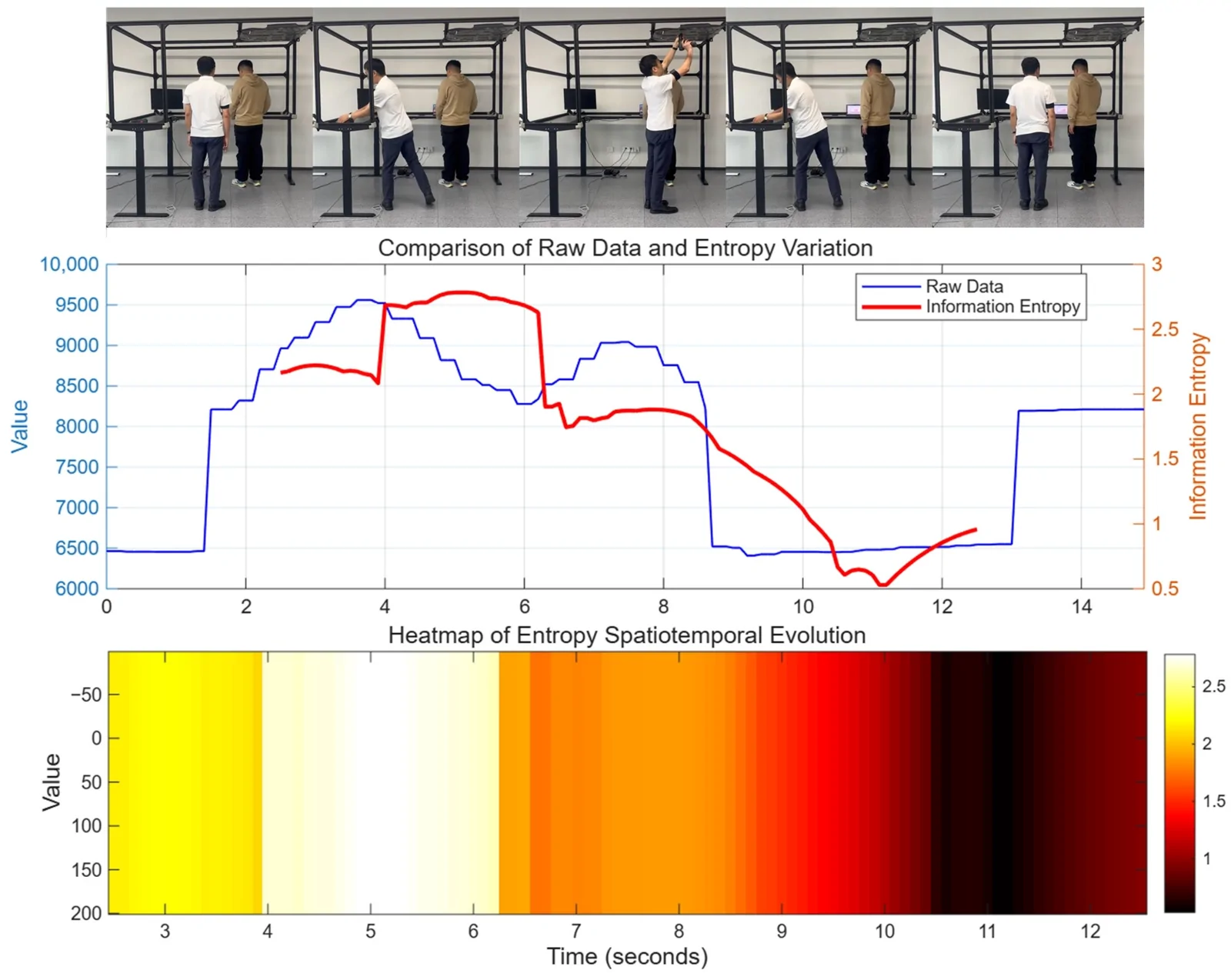
The experimental operation process and the evolution of entropy.

To ensure the accuracy and comparability of signal analysis results, unified and standardized preprocessing was performed on the original interface pressure signals in this experiment, mainly including three steps: invalid data processing, signal denoising and correction, and multi-stage signal segmentation. The specific procedures are as follows: (1) Invalid data elimination and time alignment. The unstable and invalid signals in the first 3 s action-transition stage of the experiment were eliminated. All valid signals were uniformly aligned to the time zero point to eliminate the time offset caused by operation-start delay and ensure the time sequence consistency of data. (2) Filtering denoising and baseline correction. A 5 Hz low-pass filter was used to remove high-frequency noise and correct baseline drift synchronously. On the basis of effective interference removal, the core dynamic characteristics of interface pressure signals were retained to the maximum extent to ensure the authenticity and integrity of signals. (3) Accurate multi-stage segmentation. Based on the five preset core operation stages, the preprocessed complete signals were accurately segmented to obtain standardized segmented signal samples. Figure 5 illustrates the experimental operation process and the evolution of entropy.

During the resting phase, signal complexity remains at a high level, as muscles stay in a naturally relaxed baseline state with prominent signal irregularity and randomness. Upon entering the drill-grasping phase, increased synergistic muscle activation improves signal regularity and reduces complexity to a medium–high level. In the core operation phase, sustained muscle contraction generates maximum torque output and stabilizes neuromuscular control, with signal complexity reaching its peak throughout the entire process. After external load removal in the drill-releasing phase, muscle control gradually recovers dynamic fluctuation characteristics, and complexity falls to a medium level. In the final standing recovery phase, complexity returns to medium–high values but still fails to fully revert to the initial resting baseline. Combined with the heatmap visualization, the gradual rigidity of upper limb muscle control can be clearly observed. Although the entropy value partially recovers in the later stage, the overall fatigue level continues to increase, which demonstrates the core physiological implications of information entropy: it quantifies the spatial dispersion of interface pressure amplitude and effectively characterizes the progressive deterioration of muscle contraction stability caused by cumulative fatigue during long-term overhead operation.

### 4.2. Result Analysis

**Figure 6:**
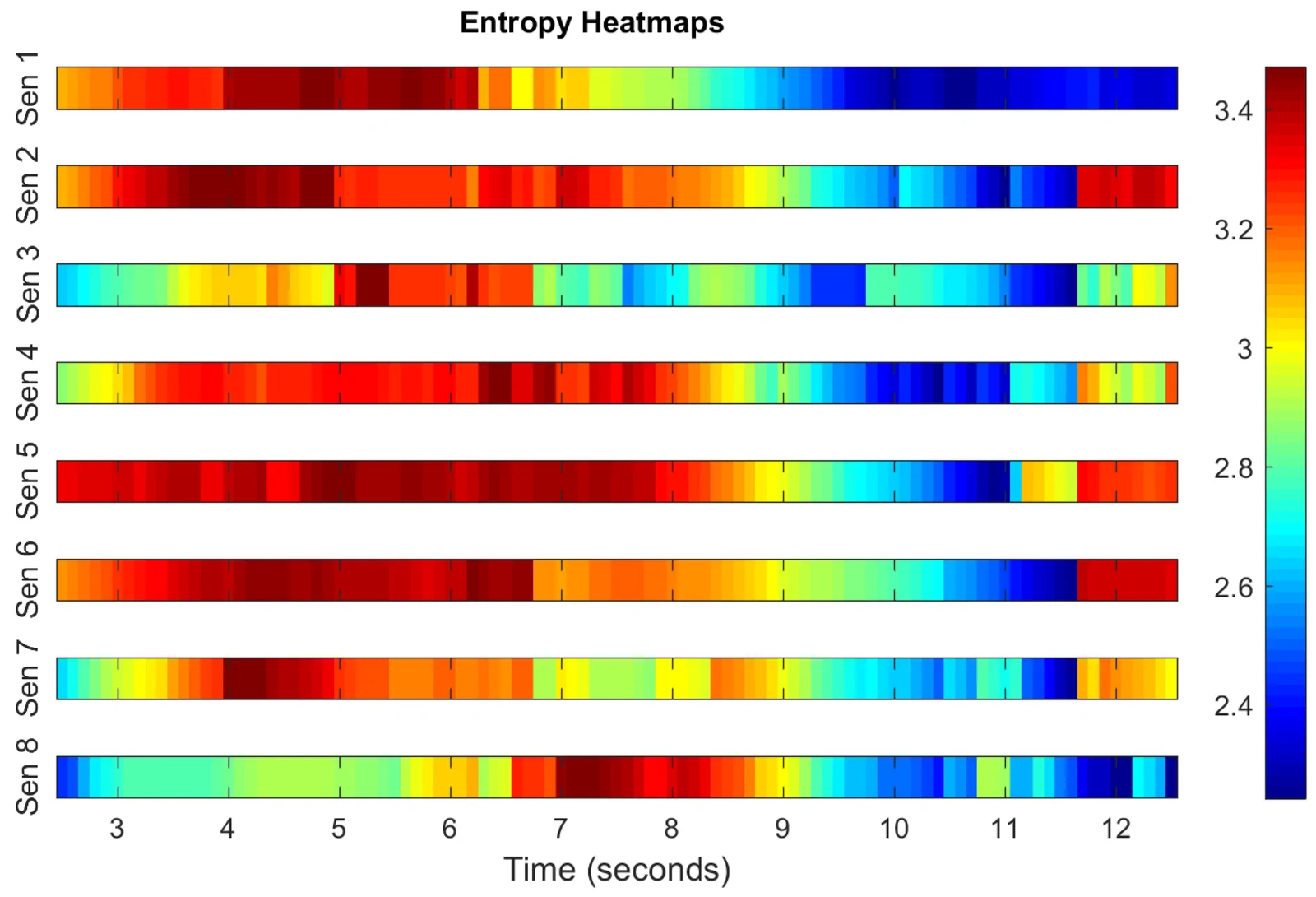
The entropy heatmaps of the muscle interface pressure.

Upper limb fatigue triggered by overhead operations deteriorates muscle contraction stability and disturbs biomechanical states, which can be quantitatively characterized by the spatiotemporal features of interface pressure signals; as spatial and temporal characteristic parameters, information entropy and sample entropy reflect fatigue generation and accumulation in different dimensions, supporting hierarchical and comprehensive fatigue analysis. Figure 6 illustrates the entropy heatmaps of the muscle interface pressure.

**Figure 7:**
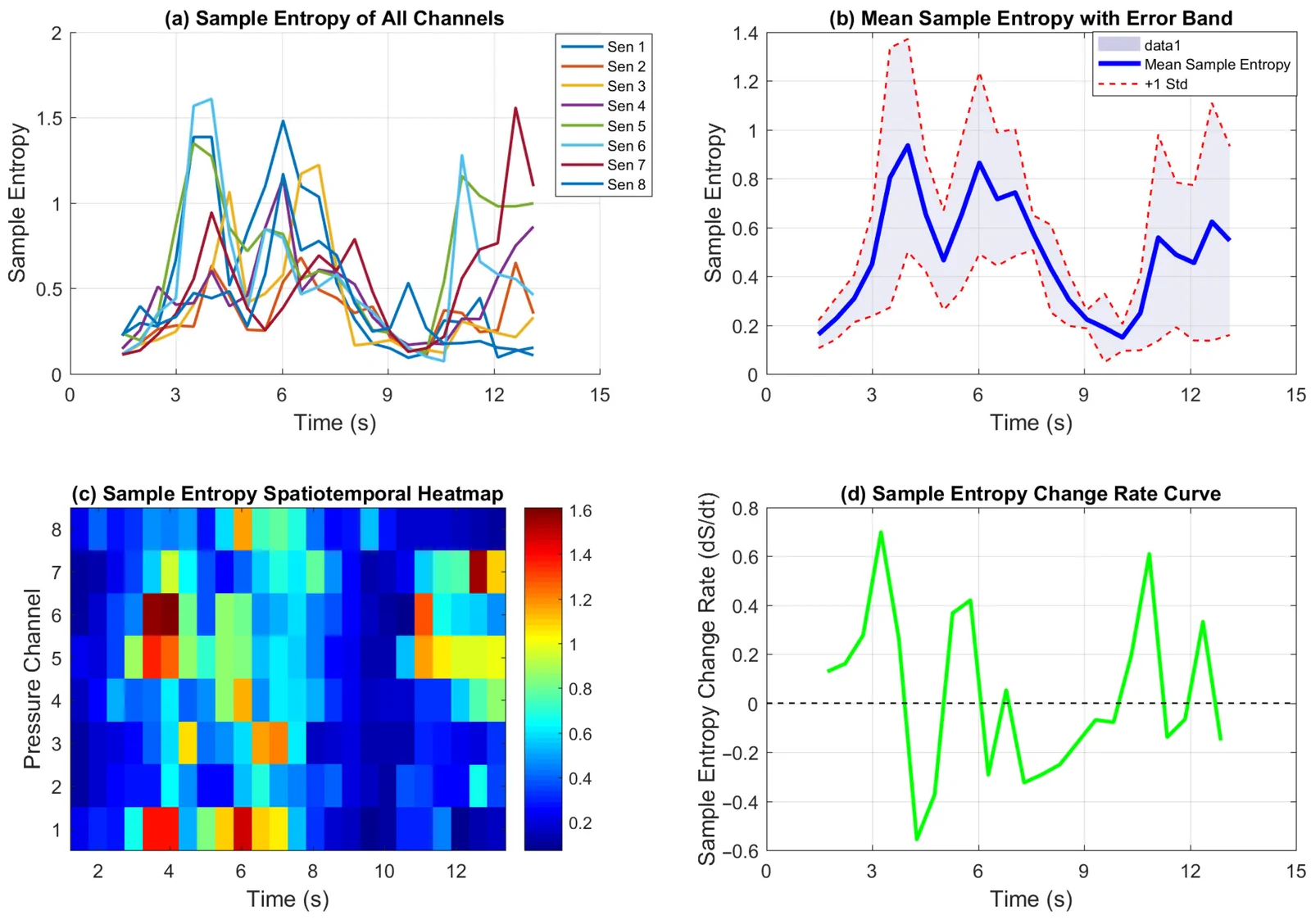
The sample entropy of the muscle interface pressure.

Information entropy quantifies the spatial dispersion of pressure amplitude and reflects the stability of muscle contraction. With the accumulation of upper limb fatigue during overhead work, the stability of muscle contraction deteriorates, and pressure fluctuation intensifies, which leads to a continuous increase in information entropy. The entropy value rises evidently from the initial stage to the late stage of operation. Channels 5 and 6 present the highest entropy values and are most sensitive to fatigue changes. Channel 3 maintains a lower entropy level with better local muscle stability. The results indicate that information entropy can serve as a reliable indicator for evaluating muscle fatigue. A growth rate exceeding 5% can effectively identify the occurrence of muscle fatigue. Figure 7 illustrates the sample entropy of the muscle interface pressure.

Sample entropy measures the temporal regularity and coordination of muscle contraction. It is highly sensitive to fatigue-induced disordered movement. Fatigue accumulation disrupts regular muscle contraction, increases temporal complexity of pressure signals, and continuously raises sample entropy. Monitoring thresholds are established according to the core fatigue characteristics of degraded muscle contraction stability and impaired muscle coordination. Within 15 s, information entropy increases by 7.1%, exceeding the predefined threshold of 5% and indicating declined muscle contraction stability. Meanwhile, sample entropy rises by 11.8%, which surpasses the 10% rate-of-change threshold and reveals aggravated muscle coordination deterioration. Compared with information entropy, sample entropy achieves a larger growth amplitude and faster response rate. Thus, the method demonstrates superior sensitivity to fatigue evolution. As a valid temporal indicator, sample entropy can reflect the degradation of muscular regularity and coordination. An increase beyond 10% can be regarded as significant muscle fatigue. As the requirements for stability and output efficiency increase, the sample entropy generally decreases. Specifically, when transitioning from static to dynamic exertion, the neuromuscular system shifts from a flexible and complex regulatory mode to a more regular low-entropy control mode. Moreover, the sample entropy also has excellent anti-interference capabilities, acting as a reliable biomarker for revealing the neural control mechanism and evaluating the level of muscle exertion. The dual-entropy variations and muscle coordination degradation are presented in Table 1.

### 4.3. Comparative Analysis of Interface Pressure and sEMG Signal

**Figure 8:**
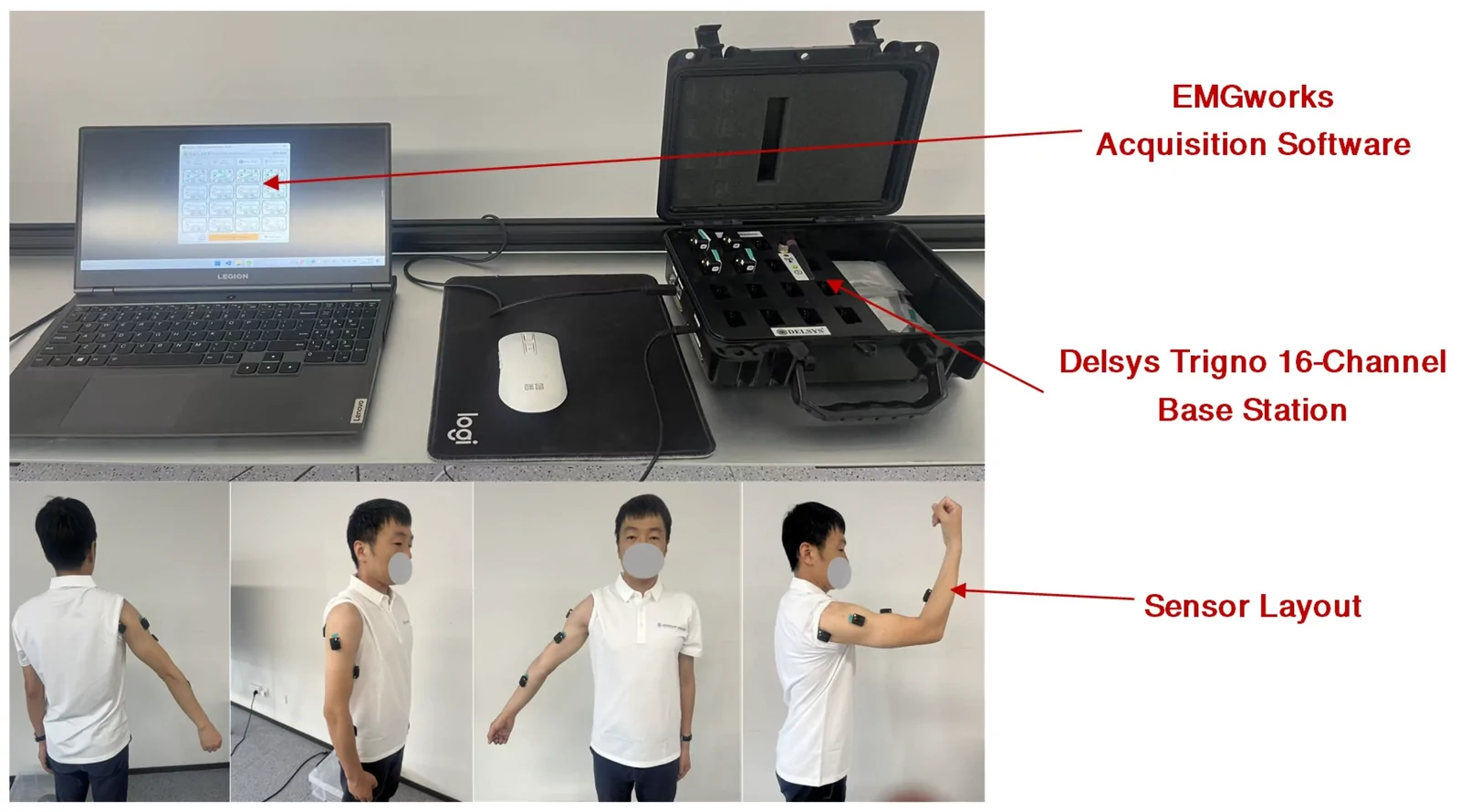
The experimental equipment and sensor layout.

**Figure 9:**
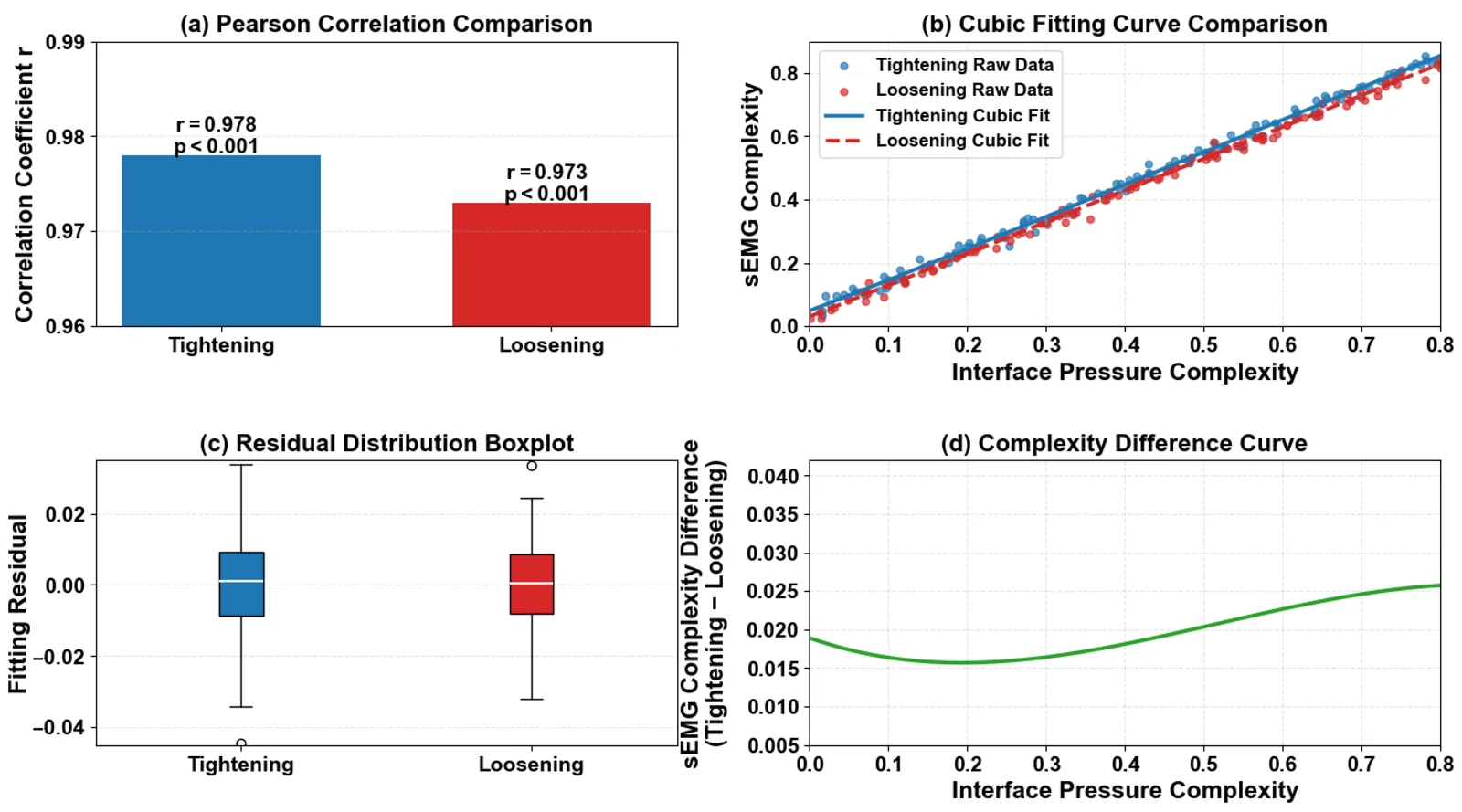
The correlation and sensitivity analysis of interface pressure and sEMG signals.

To verify the reliability and sensitivity of the interface pressure-based fatigue monitoring method, parallel experiments were conducted using sEMG as a reference. sEMG signals were collected through the Delsys Trigno 16-Channel Base Station and EMGworks Acquisition Software 4.8.0, Natick, MA, USA. The integrated sensors, equipped with sEMG electrodes and IMUs, operated at a sampling frequency of 2000 Hz. sEMG features and pressure-based dual-entropy features were compared in four dimensions: correlation significance, curve-fitting performance, residual error distribution, and cross-condition fatigue differences. The experimental equipment and sensor layout are shown in Figure 8. The correlation and sensitivity analysis of interface pressure and sEMG signals are shown in Figure 9.

Comparative evaluations concerning correlation, fitting performance, prediction accuracy, and operational difference are conducted to explore the muscle fatigue characterization capacity of interface pressure dual-entropy complexity and sEMG fatigue features during overhead tightening and loosening operations. Highly significant positive correlations exist between the two fatigue characterization approaches. The correlation coefficients are 0.978 and 0.973 for tightening and loosening tasks (*p* < 0.001), respectively, which demonstrates a tight coupling effect between external interface mechanical perturbation and internal neuromuscular fatigue response.

The cubic polynomial model presents outstanding fitting performance with a determination coefficient of R^2^ = 0.9927, effectively capturing the nonlinear evolution characteristics of muscle fatigue and addressing the inherent limitations of traditional linear fitting methods. Residual errors are densely distributed around zero, confirming high prediction accuracy and stability of the model under diverse working conditions. Tightening operations trigger more severe residual fatigue that is difficult to recover from, compared with loosening operations. The dual-modal complexity can precisely characterize the nonlinear coupling relationship between external interface pressure signals and internal upper limb muscle fatigue. The model demonstrates superior performance in fatigue quantification and nonlinear trend identification.

### 4.4. Comparative Analysis of Dual Entropy

**Figure 10:**
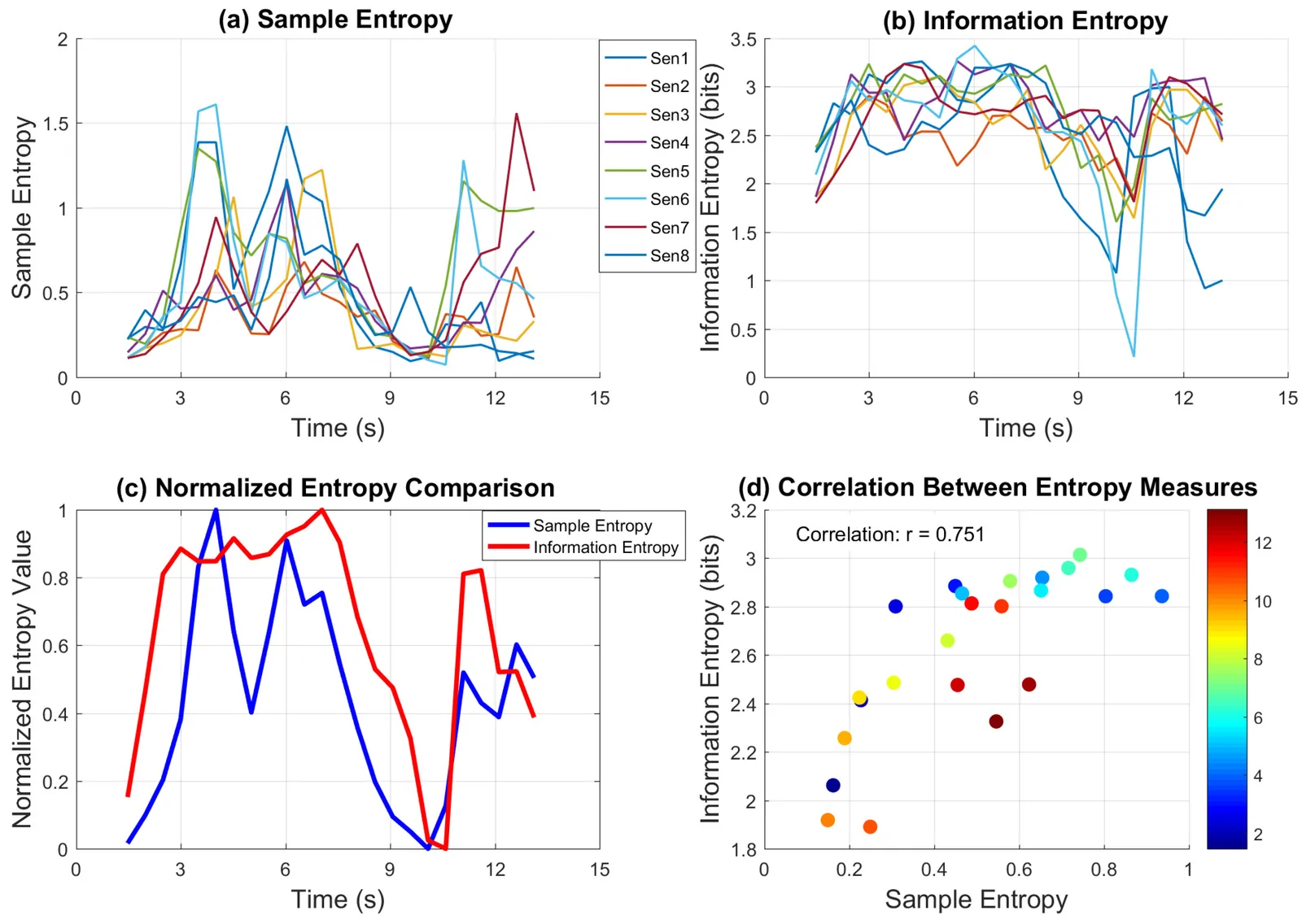
The comparative results of sample entropy and information entropy.

Sample entropy and information entropy characterize pressure signals from temporal complexity and spatial dispersion perspectives, with their complementary advantages overcoming the limitations of single-index evaluation. The synchronous growth of dual-entropy indicators accurately reflects the progressive accumulation of upper limb muscle fatigue and provides effective support for multi-dimensional fatigue identification and monitoring in overhead work. Figure 10 illustrates the comparative results of sample entropy and information entropy.

Sample entropy and information entropy possess distinct physical implications and evaluation dimensions for muscle fatigue assessment, presenting prominent complementary advantages. The correlation coefficient between the two indicators reaches 0.751, indicating partial correlation, rather than complete consistency. Sample entropy is more sensitive to dynamic changes in upper limb fatigue, focusing on analyzing the temporal characteristics of pressure signals to capture the disorder and regular deterioration of muscle movement during fatigue evolution; in comparison, information entropy ignores temporal features and exclusively characterizes the spatial dispersion variation in interface pressure amplitude. The two indicators cover the temporal and spatial dimensions of muscle fatigue, respectively, effectively eliminating the one-sidedness and dimensional limitations of single-index evaluation.

This study further constructs a hierarchical fatigue monitoring method based on the dual-entropy experimental thresholds, endowing the two indicators with differentiated monitoring functions. Information entropy is applicable to early fatigue warning, while sample entropy is dedicated to identifying obvious fatigue status. In practical overhead operation scenarios, abnormal fluctuation of a single indicator signals slight early fatigue, which can be alleviated by adjusting working postures. In contrast, synchronous abnormal growth of dual-entropy indicators denotes severe neuromuscular fatigue, requiring immediate work suspension and rest to block persistent fatigue accumulation.

During continuous overhead operations, both entropy indicators show steady upward trends, demonstrating the increased complexity of neuromuscular movement and the progressive accumulation of upper limb fatigue. In the initial operation stage, interface pressure is evenly distributed with stable temporal variation. As the operation continues, interface pressure fluctuations become disordered, and the spatial dispersion of pressure amplitude increases gradually. The synchronous variation in the two indicators verifies the inherent correlation between the temporal complexity and spatial distribution characteristics of interface pressure, fully confirming the feasibility and practicality of the dual-entropy fusion strategy for dynamic muscle fatigue monitoring in overhead tasks; this hierarchical monitoring method provides a reliable theoretical and practical reference for upper limb fatigue early warning and occupational safety protection.

## 5. Conclusions

This work develops a spatiotemporal dual-entropy fusion method for upper limb fatigue monitoring during overhead assembly operations. In contrast to conventional single-index evaluation, the proposed method integrates information entropy and sample entropy for comprehensive fatigue characterization in the spatial stability and temporal coordination dimensions. Information entropy quantifies the spatial dispersion of interface pressure amplitude for early fatigue recognition with a 5% rate-of-change threshold, while sample entropy reflects temporal muscle coordination degradation for significant fatigue identification, with a 10% rate-of-change threshold. With higher sensitivity and better anti-interference performance, the dual-entropy indicators grow synchronously to reflect progressive neuromuscular fatigue accumulation. A hierarchical monitoring strategy is established to distinguish slight and severe fatigue states; by adopting anti-interference interface pressure signals, instead of vulnerable sEMG sensors, the proposed method realizes stable, real-time, graded fatigue assessment, providing a reliable nonlinear monitoring tool for ergonomic analysis and occupational safety protection in scenarios of overhead work.

## Figures and Tables

**Table 1:** Dual-entropy variations and muscle coordination degradation.

Identification Indicators	Fatigue Characteristics	15 s Rate of Change	Identification Threshold
Information entropy	Muscle contraction stability decreases	↑ 7.1%	>5%
Sample entropy	Muscle coordination deteriorates	↑ 11.8%	>10%

## Data Availability

The data presented in this study are available on request from the corresponding author.

## Abbreviations

The following abbreviations are used in this manuscript:

UHV	Ultra-High Voltage
sEMG	Surface Electromyography
MMG	Mechanomyography
LZ	Lempel–Ziv
MPF	Mean Power Frequency
WMSDs	Work-Related Musculoskeletal Disorders
EEG	Electroencephalogram
MCI	Muscle Contraction-Induced
FSR	Force-Sensitive Resistor
IMU	Inertial Measurement Unit
SOPs	Standard Operating Procedures

## Appendix A

**Algorithm A1.** Information entropy calculation
**Input**: Time series data *X* = [*x*1, *x*2, …, *x*N], Number of bins *B* **Output**: Information entropy value *H* **1.** Initialize: Calculate min_val = min(*X*) Calculate max_val = max(*X*) Calculate bin_width = (max_val − min_val)/*B* **2.** Create bins and count: Initialize counts [1..*B*] = 0 **FOR** *i* = 1 TO N: bin_index = FLOOR((*X*[*i*] − min_val)/bin_width) + 1 **IF** bin_index > *B*: bin_index = *B* **END IF** counts[bin_index] = counts[bin_index] + 1 **END FOR** **3.** Calculate probability distribution: **FOR** *i* = 1 **TO** *B*: prob[*i*] = counts[*i*]/N **END FOR** **4.** Calculate information entropy: *H* = 0 **FOR** *i* = 1 TO *B*: **IF** prob[*i*] > 0: *H* = *H* − prob[*i*] * log2(prob[*i*]) **END IF** **END FOR** **5.** RETURN *H*

**Algorithm A2.** Sample entropy calculation
**Input:** Time series data *X* = [*x*1, *x*2, …, *x*N], Embedding dimension *m*, Tolerance *r* **Output:** Sample entropy value SampEn **1.** Define template matching function count_matches(*X*, *m*, *r*): *N* = length(*X*) count = 0 **FOR** *i* = 1 **TO** *N* − *m*: **FOR** *j* = *i* + 1 **TO** *N* – *m* + 1: Calculate Chebyshev distance: dist = max (|*X*[*i*:*i* + *m* − 1] − *X*[*j*:*j* + *m* − 1]|) **IF** dist < *r*: count = count + 1 **END IF** **END FOR** **END FOR** RETURN count **2.** Calculate match counts: B = count_matches(*X*, *m*, *r*) A = count_matches(*X*, *m* + 1, *r*) **3.** Calculate sample entropy: **IF** *B* > 0 AND *A* > 0: SampEn = − ln(*A*/*B*) **ELSE**: SampEn = NaN **END IF** **4.** RETURN SampEn
